# Current-Resistance Effects Inducing Nonlinear Fluctuation Mechanisms in Granular Aluminum Oxide Nanowires

**DOI:** 10.3390/nano10030524

**Published:** 2020-03-14

**Authors:** Carlo Barone, Hannes Rotzinger, Jan Nicolas Voss, Costantino Mauro, Yannick Schön, Alexey V. Ustinov, Sergio Pagano

**Affiliations:** 1 Dipartimento di Fisica “E.R. Caianiello”, Università degli Studi di Salerno, I-84084 Fisciano, Salerno, Italy; cmauro@unisa.it (C.M.); spagano@unisa.it (S.P.); 2 CNR-SPIN Salerno, c/o Università degli Studi di Salerno, I-84084 Fisciano, Salerno, Italy; 3 INFN Gruppo Collegato di Salerno, c/o Università degli Studi di Salerno, I-84084 Fisciano, Salerno, Italy; 4 Physikalisches Institut, Karlsruher Institut für Technologie, 76131 Karlsruhe, Germany; rotzinger@kit.edu (H.R.); jan.voss@kit.edu (J.N.V.); y.schoen@kit.edu (Y.S.); alexey.ustinov@kit.edu (A.V.U.); 5 Institut für Quantenmaterialien und Technologien (IQMT), Karlsruher Institut für Technologie, 76131 Karlsruhe, Germany; 6 National University of Science and Technology MISIS, 119049 Moscow, Russia; 7 Russian Quantum Center, Skolkovo, 143025 Moscow, Russia

**Keywords:** quantum electronics, noise spectroscopy, granular aluminum oxide, superconducting nanowires, current-resistance effects

## Abstract

The unusual superconducting properties of granular aluminum oxide have been recently investigated for application in quantum circuits. However, the intrinsic irregular structure of this material requires a good understanding of the transport mechanisms and, in particular, the effect of disorder, especially when patterned at the nanoscale level. In view of these aspects, electric transport and voltage fluctuations have been investigated on thin-film based granular aluminum oxide nanowires, in the normal state and at temperatures between 8 and 300 K. The nonlinear resistivity and two-level tunneling fluctuators have been observed. Regarding the nature of the noise processes, the experimental findings give a clear indication in favor of a dynamic random resistor network model, rather than the possible existence of a local ordering of magnetic origin. The identification of the charge carrier fluctuations in the normal state of granular aluminum oxide nanowires is very useful for improving the fabrication process and, therefore, reducing the possible sources of decoherence in the superconducting state, where quantum technologies that are based on these nanostructures should work.

## 1. Introduction

Granular superconductors have attracted great interest for their rich phase diagram and for practical advantages, such as increased critical field [1], critical temperature [2,3], and kinetic inductance [4]. In particular, granular aluminum oxide (AlO_x_) has recently proven to play a prominent role in quantum electronics and in quantum bit (qubit) design due to its low electric loss and high kinetic inductance [5]. As a matter of fact, the kinetic inductance of an AlO_x_ wire is proportional to its normal state resistance and, therefore, can be orders of magnitude higher than its geometric inductance [6]. Such a super-inductance could be very useful for the realization of a novel type of superconducting quantum circuit with an impedance above the vacuum impedance, and it could easily be constructed from AlO_x_ wires [5,7]. The robustness of the material and the simplicity of fabrication make them a promising candidate to replace conventional Josephson tunnel junction arrays. The main differences with Josephson junctions are observed when AlO_x_ is patterned in the form of a nanowire. The nanoscale structure can be advantageous in terms of electric loss, and in the limit of a short constriction can even be used as a non-linear element for quantum circuit design [8].

Although granular aluminum seems to be a very promising alternative material for qubit architecture, it has a disordered nature, due to a large amount of amorphous aluminum oxide. Previous published structural and morphological studies suggest that the granularity on the size of grains, connectedness, and the intergrain spacing depend on the preparation method [9], which results in films with a disorder distributed on a scale that ranged from few units to few tens of nanometers [10,11]. These types of granular films have different electric transport mechanisms, whose understanding is important, especially for systems realized at the nanoscale level. In this respect, noise spectroscopy has already been used for the sensitive investigation of low-dimensional superconducting films [12] and two-dimensional (2D) oxide interfaces [13,14], allowing for a better understanding of the charge carriers kinetic processes. Moreover, from a technological point of view, the integration of nanowire-based elements with standard circuit processing can be seriously limited by the increase of the 1/f electric noise. Therefore, such a noise component has to be carefully studied in order to have a deep comprehension of the nature of fluctuation mechanisms.

In view of all these considerations, DC electric and magneto-transport measurements, as well as voltage-noise investigations, have been performed on AlO_x_ nanowires. The observed phenomenology, although related to the normal state, deserves to be understood in details, since it could also have an impact on the superconducting state, where qubit-based technology operates.

## 2. Materials and Methods

All of the investigated wires, as well as the surrounding structures, were made from a single layer of oxidized aluminum. As a first step, a 20 nm thick layer of disordered oxidized (granular) aluminum was grown by reactive sputter deposition on a *c*-plane sapphire substrate in a controlled oxygen atmosphere, thus allowing for a wide range of sheet resistances. The mean sheet resistance of the AlO_x_ film was determined to be about 2.2 kΩ. However, the sheet resistance can vary up to 50% for different film areas across a 20 mm by 20 mm chip due to inhomogeneities that are related to the sputtering process.

An electron beam lithography process was used to define the wire structures, while using a bilayer of hydrogen silsesquioxane (HSQ) and polymethylmethacrylate (PMMA) resist. Here, the high resolution of the HSQ and the ability to use an organic solvent to lift off the PMMA are beneficial for a clean interface and also to contact the AlO_x_ structures. The PMMA serves as a sacrificial layer, it, however, also influences (limits) the achievable nanowire resolution. Two anisotropic dry etch (RIE) steps were performed to transfer the structures into the AlO_x_ layer after the resist definition: oxygen RIE for the PMMA, and argon/chlorine RIE for the AlO_x_.

**Figure 1:**
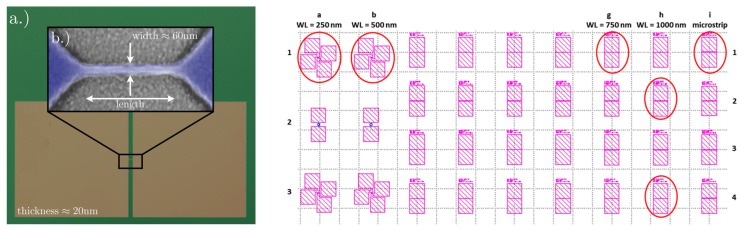
(Left panel) Colorized optical picture of a device investigated. The colorized scanning electron microscope image of a typical nanowire is shown in the enlargement. The wire is 20 nm thick and it has a typical width up to 60 nm. (Right panel) Layout of the chip. The measured nanowire samples are evidenced by the circles. The contacts are made by 50 μm thick with aluminum wire by ultrasonic bonding.

The left panel of Figure 1 shows a typical scanning electron microscope image of another nanowire sample that was fabricated with similar process conditions. Here, the wires have a thickness of 20 nm and a width of few tens of nanometers, in the order of 60 nm. It has been found that small variations in the process parameters can lead to a significant spread in the wire width and resolution of the above described bilayer process. We suspect that the limited temperature stability of the PMMA resist might be responsible for this and, however, further optimizations may be necessary for a better reproducibility of the nanowire shape. Several devices were measured, each of them made by two 150 μm × 150 μm contact pads connected by a narrow stripline, as shown in the right panel of Figure 1. Nanowires of different lengths are fabricated in the middle of each stripline. Devices, named “a”, “b”, “g”, and “h” are the nanowires of length (*WL*) 250, 500, 750, and 1000 nm, respectively. Devices named “i”, instead, are microstrips 5 μm-long and, nominally, 100 nm-wide.

A simple two-probe measurement method was employed, since all of the measured resistances were much higher than that of the contact wires to investigate the samples transport and noise properties. Additionally, the wires and contacts contribution to the overall electric noise was verified to be negligible [15]. The value of resistance measured involves the whole stripline, not only the submicron part. This implies that, for devices “a”, “b”, “g”, and “h”, there are additionally 10 squares contributions to consider for the total resistance. Electric transport measurements were performed in a temperature stabilized closed-cycle refrigerator, between 300 and 8 K. A dipole electromagnet, type 3470 from GMW Associates, generated an external magnetic field (maximum value of 1500 Gauss). The field is applied in the plane of the sample and parallel to the nanowire length which coincides with the bias current direction. Low-noise DC and AC electronic bias and readout have been used [15,16], thus giving very low statistical errors on the experimental data points (in the following plots, the error bars are usually smaller than the points dimension). The range of explored bias currents depends on the sample resistance, being limited by the overall voltage drop that is available at the current source (100 V).

## 3. Results

### 3.1. DC Electrical Transport Measurements

**Figure 2:**
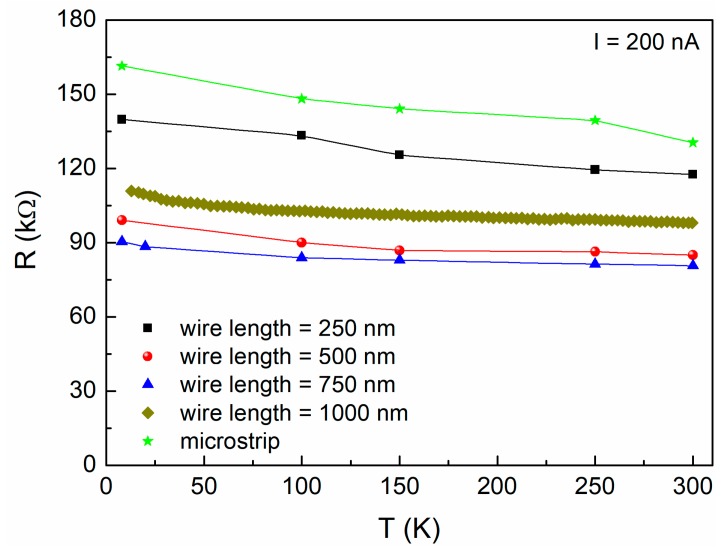
Resistance versus temperature plots. The data refer to five investigated devices. The solid lines are obtained by interpolating the experimental points and they are guides for the eyes.

Figure 2 shows the temperature dependence of the resistance of all the investigated devices. Differently from the previously analyzed case of AlO_x_ thin films [17], the nanowires are characterized by a monotonic resistivity decrease by increasing the temperature between 8 and 300 K. This decreasing behavior, which is not unusual in high resistive samples, seems to be more pronounced at low temperatures, although the resistance value does not scale with the wire length, probably due to differences of the average wire width and resistivity across the nanowire devices. By using the microstrip (nominally 100 nm-wide) as a reference, it is possible to estimate the room-temperature resistivity of the nanowires *ρ_RT_* ≈ 5.2 × $10^{3}$ μΩ cm. For these values of *ρ_RT_* (higher than 4 × $10^{3}$ μΩ cm), the granular aluminum specimens are characterized by a resistance that rises monotonically as the temperature is reduced, confirming the experimental behavior shown in Figure 2, as reported in literature [18,19]. Conversely, for *ρ_RT_* values between 1 × $10^{3}$ and 4 × $10^{3}$ μΩ cm there is an intermediate regime in which the resistance changes very little with temperature and it shows a logarithmic increase at low temperatures (Kondo regime). This is the case of the previously analyzed AlO_x_ thin films [17], which, therefore, have a resistivity temperature dependence different from the nanowires studied here. It is also important to underline that the strictly metallic regime is usually characterized by values of *ρ_RT_* that are lower than $10^{3}$ μΩ cm [20].

**Figure 3:**
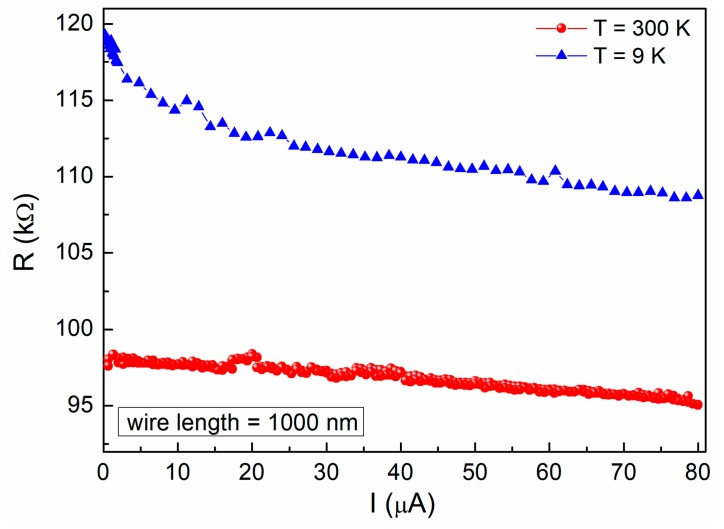
Static resistance versus bias current at different temperatures. The data refer to the nanowires 1000 nm-long at *T* = 300 K (red dots) and at *T* = 9 K (blue triangles). The small jumps visible in the traces are reproducible and are, most probably, due to different current paths in the sample.

All of the devices exhibit almost linear current voltage characteristics, with a small curvature that can be observed for bias current above few μA. By analyzing in detail the bias current dependence of the static resistance *R*, defined as the ratio *V*/*I*, the wire resistance shows a tendency to decrease for increasing bias. The repeatability of this effect has been verified in all of the nanostructures analyzed, by performing several current cycles from 0 to 80 μA. Consequently, the reversible resistance change is independently present from the temperature, but is more pronounced at low temperatures, as shown in Figure 3. This has been also observed on manganite compounds and it is known as the negative current-resistance (CR) effect [21,22,23,24]. Several mechanisms could be considered to explain the experimental evidence of reversible CR effects in AlO_x_ nanowires. In analogy with the case of magnetic multilayered structures [25,26], the CR effect could be attributed to a local ordering that is produced by a current-induced local magnetic field. Alternatively, the CR effect could be ascribed to a percolative process between metallic AlO_x_ clusters that are separated by an insulating matrix [27,28]. In this framework, an important role is played by the tunneling barrier between conducting grains and by the shape of such a barrier, which the applied current can change.

Overall, DC measurements alone are not able to distinguish the possible mechanism that is responsible for the CR effect. Therefore, more sensitive investigations, such as electric noise spectroscopy, have been performed, in order to acquire additional information.

### 3.2. Voltage-Noise Spectral Density Measurements

**Figure 4:**
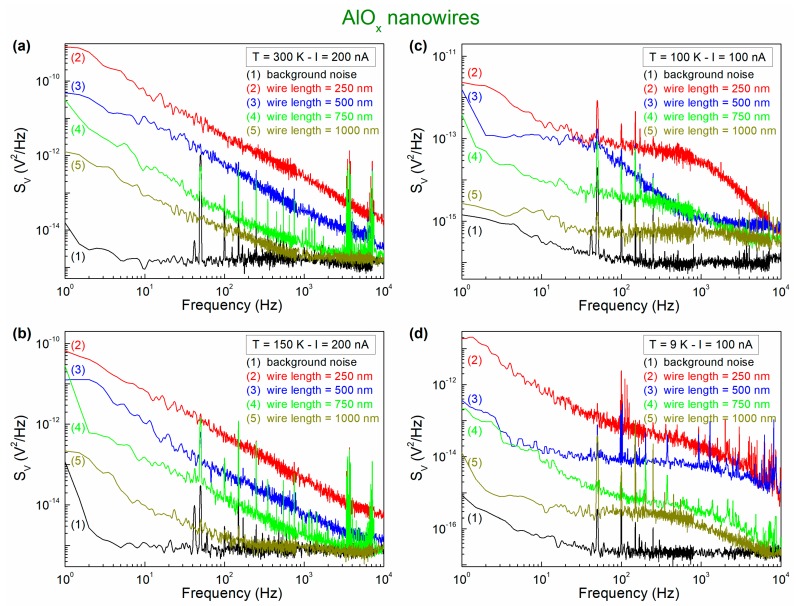
Voltage-noise spectra. The frequency dependence of *S_V_* is shown for all of the investigated nanodevices for two bias currents and four different temperatures: 300 K (**a**), 150 K (**b**), 100 K (**c**), and 9 K (**d**). The black trace, which is the lowest spectral trace for each temperature, shows the unbiased noise, which is composed by the instrumental, the external, and the sample Johnson noise.

The first indication on the nature of the fluctuation processes is obtained by analyzing the frequency composition of the voltage-noise spectral density *S_V_*. In Figure 4, typical noise spectral traces are reported for the four nanowires under test at different bias current values and temperatures. The peaks are due to external sources and they should not be considered. The relevant information is in the background curves. *S_V_* is characterized by a frequency dependence that varies on temperature. In particular, the presence of a 1/*f* noise component followed by a constant amplitude spectrum is observed at temperatures down to 150 K (Figure 4a,b). Conversely, below 150 K, Lorentzian components, with a 1/*f* ^2^ dependence of *S_V_*, are clearly visible (Figure 4c,d). This behavior is typically associated with physical fluctuation mechanisms that are ascribed to two-level tunneling fluctuators (TLTFs) [29,30]. According to this interpretation, groups of atoms can occupy two configurations; therefore, their energy can be represented in the form of two potential wells that are separated by a barrier. In general, the wells are asymmetric and the atoms can tunnel from one well to the other with a transition rate that is strongly temperature-dependent. By indicating with *τ* the minimum relaxation time of such processes, for frequencies f≪1/2πτ the main contribution to *S_V_* is given by the 1/*f* noise, while, for frequencies f≫1/2πτ, the spectral density is dominated by Lorentzian noise. By lowering the temperature, *τ* increases and the cut-off frequency, above which the 1/*f* ^2^ noise component dominates, decreases, being visible inside the frequency bandwidth of acquisition (see, for details, all of the low-temperature spectra in Figure 4).

**Figure 5:**
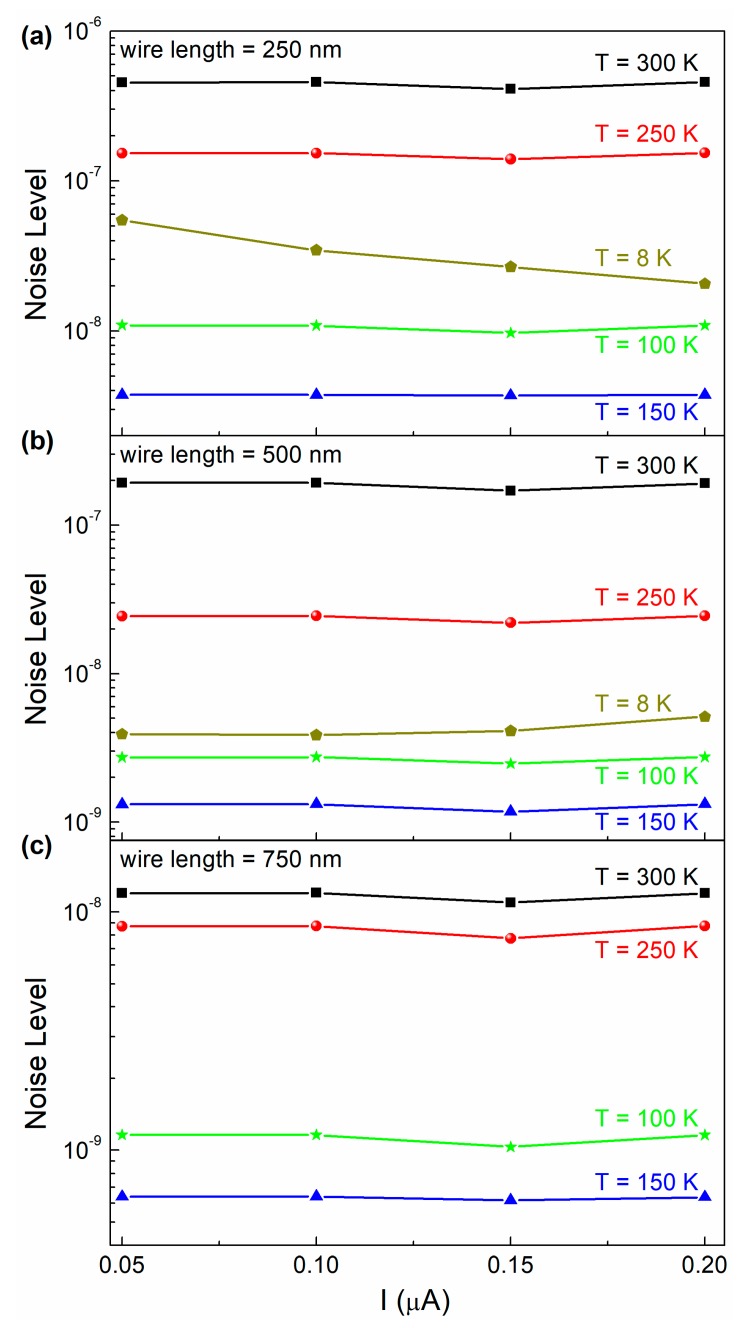
Current dependence of the nanodevices noise level in the low-bias region. The noise level dependence on the bias current, as evaluated from Equation (1), is shown for currents up to 200 nA and for devices of different lengths: 250 nm (**a**), 500 nm (**b**), and 750 nm (**c**). The investigated temperature range is from 8 to 300 K.

Spontaneous transitions between the levels of the TLTFs can lead to fluctuations of macroscopic quantities, such as resistance [29]. In the ohmic region, where resistance does not depend on the bias current, resistance fluctuations are characterized by a *S_V_* versus *I* ^2^ dependence. This means that the overall noise level, being defined as:

(1) $$Noise\;Level=\frac{1}{V^{2}}\int_{f_{min}}^{f_{max}}S_{V}\left(f\right)df,$$

with [*f_min_* ; *f_max_*] as the frequency bandwidth, should be constant as a function of the bias current. A confirmation of this behavior is found in the plots of Figure 5, where the current dependence of the noise level is shown for three devices at different temperatures, for low levels of the applied bias. In Figure 5, it is also evident a noise level increase when the temperature is reduced below 150 K. This is due to the activation of additional low-temperature noise sources, being identified in the framework of the TLTFs model and evidenced in the spectral traces as Lorentzian components (see Figure 4c,d, for details).

**Figure 6:**
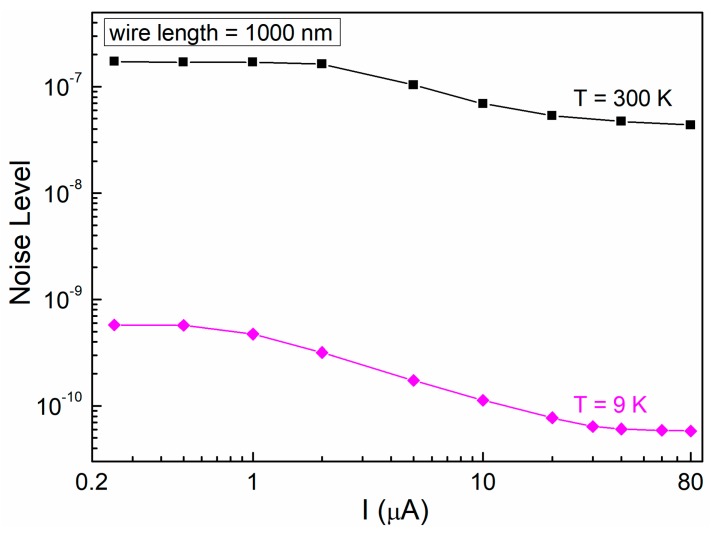
Current dependence of the nanodevices noise level in the full-bias region. The overall noise level, as evaluated with Equation (1), is shown as a function of the bias current for the nanowire 1000 nm-long. The temperatures of 300 and 9 K are reported.

However, for bias currents where CR effects are present, the noise level also changes. In particular, the data in Figure 6 show a noise level reduction at large levels of the applied bias. This behavior, which is evident both at room temperature and at 9 K (black squares and magenta diamonds in Figure 6, respectively), indicates, therefore, that the local ordering that is attributed to CR effects gives origin to a decrease of noise fluctuators. The decrease of the noise level, both for decreasing temperature and for increasing DC bias current, seems to be incompatible with a Joule heating effect. In fact, Joule heating would imply an increase of the nanowire temperature with the bias current, and this should also result in an increased noise level, since it is enhanced by temperature. Instead, Figure 6 shows clearly that the noise level decreases at larger bias currents.

The nature of the noise decrease and, consequently, the nature of reversible-type CR phenomena is at this stage unclear, but a better insight could be obtained by looking at its dependence on external parameters, such as external magnetic field or electric field.

## 4. Discussion

As said before, in other materials, it has been found that possible mechanisms producing the current-resistance effects and, at the same time, the noise level reduction could be of magnetic origin or could be due to the percolative process. Whether one of these models can be associated to granular AlO_x_ nanowires has to be investigated.

**Figure 7:**
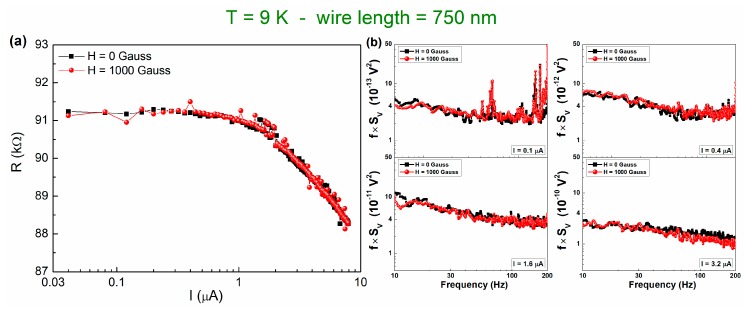
Magnetic field behavior of DC and AC data at 9 K. The current dependence of (**a**) the resistance and of (**b**) the normalized 1/f noise component of device 750 nm-long is plotted in absence and in presence of a magnetic field of 1000 Gauss (black and red curves, respectively).

Magnetic resistance fluctuations are usually characterized by a noise level increase in the low-temperature region. This type of fluctuation process, which is already found for granular aluminum oxide thin films, is ascribed to Kondo-like conductivity when the noise level, especially at low temperatures, decreases with an external magnetic field of the order of one thousand Gauss [17,31]. In the case of AlO_x_ nanowires, no magnetic dependence is observed in both DC and AC measurements, even if a noise enhancement is evident by lowering the temperature (for details, see Figure 7). By applying an external magnetic field *H* = 1000 Gauss, the magnitude of the CR effect is unaltered (Figure 7a). Moreover, the normalized 1/*f* noise power (*f*⋅*S_V_*) does not change with *H* for all of the bias currents used (Figure 7b). This experimental finding indicates that, contrarily to what observed for the films, in AlO_x_ nanowires, a local ordering of magnetic origin does not produce the noise reduction and, therefore, does not seem to be responsible of the measured CR effect. The different behavior that was observed between granular aluminum film and nanowires could be related to differences in morphology and grain structure in the two cases, which also show different resistivity, as discussed above.

**Figure 8:**
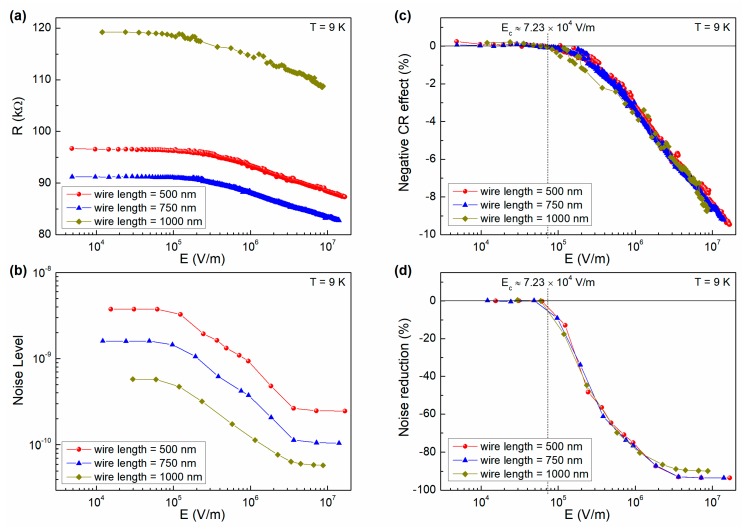
Electric field behavior of DC and AC data at 9 K. For the nanowires 500 nm-long (red circles), 750 nm-long (blue triangles), and 1000 nm-long (yellow diamonds), it is shown the electric field dependence of the: (**a**) static resistance, (**b**) noise level evaluated with Equation (1), (**c**) current-resistance effect evaluated with Equation (2), and (**d**) noise reduction evaluated with Equation (3).

Alternative to the magnetic effect, a nonlinear transport regime can also result from a random resistor network (RRN) model and, consequently, percolation processes [32,33,34]. These conduction mechanisms have been already considered in granular films that are similar to the ones investigated here [9], being realized by reactive sputter deposition and with a grain size in the order of 3–4 nm [5]. In particular, a reasonable description of nonlinear conduction is given by the dynamic random resistor network (DRRN) model, which considers a network that consists of conducting and insulating bonds [35]. For sufficiently low applied electric field across the network, the current flows only through the backbone of the percolating system, so that the conduction is linear. As the applied bias is increased, some of the insulating bonds of the network will experience an electric field exceeding a critical field (*E_c_*), above which they become conducting [35]. The macroscopic conduction becomes nonlinear when insulating bonds start to be conducting. This is similar to what is shown in Figure 8a, where the nanowire resistance is plotted versus the local electric field *E*, being computed as the voltage difference divided by the nanowire length. In this framework, the conducting clusters forming the network backbone are separated by insulating regions of very small widths. Through such regions, it is expected that tunneling or hopping conduction will take place, thus providing extra paths for electrons. The appearance of extra parallel conduction channels with the increase in bias, which add to an existing network, does lead to a decrease in the total noise of the network, which is consistent with that observed in Figure 8b.

The noise reduction above the onset of nonlinearity has been already observed in conductor-insulator mixtures, such as the carbon-wax system, and, in terms of the DRRN model, has been explained as due to an increase in the total number of fluctuators or, equivalently, in the system size from the fluctuation point of view [36]. A better display of the nonlinear transport in AlO_x_ nanowires can be made by computing the negative reversible CR effect, as:

(2) $$CR\;effect\;\left(\%\right)=\frac{R\left(E\right)-R_{0}}{R_{0}},$$

where *R*(*E*) is the resistance at a fixed electric field and *R_0_* is the resistance value in the linear region. The quantity that is defined with Equation (2) is plotted in Figure 8c as a function of *E* for three nanodevices of different length at the temperature of 9 K. Here, it is possible to identify a nonlinearity threshold at a critical electric field *E_c_* ≈ 7.2 × $10^{4}$ V m^−1^. Similarly, the noise reduction in percentage can be computed as:

(3) $$NR\;\left(\%\right)=\frac{NL\left(E\right)-NL_{0}}{NL_{0}},$$

where *NL*(*E*) is the noise level value at a fixed electric field and *NL_0_* is the noise level value in the linear region. *NR* is shown in Figure 8d for the same nanodevices of Figure 8c and it confirms the existence of a threshold field. This effect has been well documented at 9 K, and is present also at higher temperatures, see Figure S1 (Supplementary Materials). However, further analysis of the temperature dependence of the high current behavior of the nanowires has not been performed, being beyond the scope of this work, as the main applications of AlO_x_ nanowires are at cryogenic temperatures. The noise reduction effect is much more evident than the CR effect, revealing a greater sensitivity of the noise spectroscopy with respect to the resistivity measurements. Moreover, the evidence of a saturated noise level at high bias values finds strict analogy with the saturated transport properties that were observed in carbon-wax composites and described in terms of the DRRN theoretical approach [36]. This gives a further indication on the applicability of this model to explain the reversible nonlinear transport regime in AlO_x_ nanowires.

Although a nonlinear conducting behavior is expected for metal-dielectric composite nanostructures falling around the percolation threshold [37,38], the strong effect on the magnitude of voltage-noise is a peculiar feature of AlO_x_ nanowires, not being observed in common nonlinear composites dielectrics. This property of the charge carriers fluctuation mechanisms has a direct impact on the normal state conductance and, as a consequence, on the kinetic inductance of granular aluminum nanodevices, a key requirement for their use in superconducting quantum circuits.

## 5. Conclusions

The electric transport and voltage-noise properties of granular aluminum oxide nanowires have been investigated for nanodevices of different width and length and in a temperature range from 8 to 300 K. Differently from thin films, which show a linear ohmic regime, nanowires are characterized by a resistivity reduction with increasing bias. In cycled devices, the negative resistance change is reversible. In this regime, above a critical electric field, the nonlinear current-voltage characteristics are also associated with a strong noise level reduction, whose amplitude exceeds that observed on DC resistance of more than one order of magnitude. This nonlinear electric transport behavior of the nanowires does not seem to have a magnetic origin. Moreover, the magnetic field does not affect the noise amplitude, contrarily to what found in thin films with lower resistivity and at low temperatures. The nonlinear resistance could be explained, instead, in terms of a dynamic random resistor network model, usually considered as a variant of the standard random percolation mechanism. If a DRRN model describes the physics of the AlO_x_ nanowires, then it is expected that noise sources can be activated whenever there is an electric field in the material higher than a threshold critical value, and large enough fields can suppress it. In the superconducting state, one could expect that *E* field is zero inside the nanowire and no noise sources are activated. However, in the presence of RF fields, this is no longer true and the noise sources could play an important role in the dynamics of the circuit attached to these devices. Therefore, the identification and reduction of the charge carrier fluctuations in the normal state, by e.g. improving the fabrication process, could be important for reducing the possible sources of decoherence in the superconducting state.

## Supplementary Materials

The following are available online at https://www.mdpi.com/2079-4991/10/3/524/s1, Figure S1: Electric field behavior of resistance and noise data at different temperatures.
