# Supplementary material for: Current-Resistance Effects Inducing Nonlinear Fluctuation Mechanisms in Granular Aluminum Oxide Nanowires
Source: Nanomaterials (Basel). 2020 Mar 14;10(3):524. doi: 10.3390/nano10030524 (PMC7153260; doi:10.3390/nano10030524)
Supplement: Supplementary file 1 [file nanomaterials-10-00524-s001.pdf]

Supplementary Materials

Current-Resistance Effects Inducing Nonlinear Fluctuation Mechanisms in Granular Aluminum Oxide Nanowires

Carlo Barone [1,2,3,*], Hannes Rotzinger [4,5], Jan Nicolas Voss [4], Costantino Mauro [1], Yannick Schön [4], Alexey V. Ustinov [4,6,7] and Sergio Pagano [1,2,3]

[1] Dipartimento di Fisica "E.R. Caianiello", Università degli Studi di Salerno, I-84084 Fisciano, Salerno, Italy; cmauro@unisa.it (C.M.); spagano@unisa.it (S.P.)
[2] CNR-SPIN Salerno, c/o Università degli Studi di Salerno, I-84084 Fisciano, Salerno, Italy
[3] INFN Gruppo Collegato di Salerno, c/o Università degli Studi di Salerno, I-84084 Fisciano, Salerno, Italy
[4] Physikalisches Institut, Karlsruher Institut für Technologie, 76131 Karlsruhe, Germany; rotzinger@kit.edu (H.R.); jan.voss@kit.edu (J.N.V.); y.schoen@kit.edu (Y.S.); alexey.ustinov@kit.edu (A.V.U.)
[5] Institut für Quantenmaterialien und Technologien (IQMT), Karlsruher Institut für Technologie, 76131 Karlsruhe, Germany
[6] National University of Science and Technology MISIS, 119049 Moscow, Russia
[7] Russian Quantum Center, Skolkovo, Moscow 143025, Russia
* Correspondence: cbarone@unisa.it; Tel.: +39-089-968212

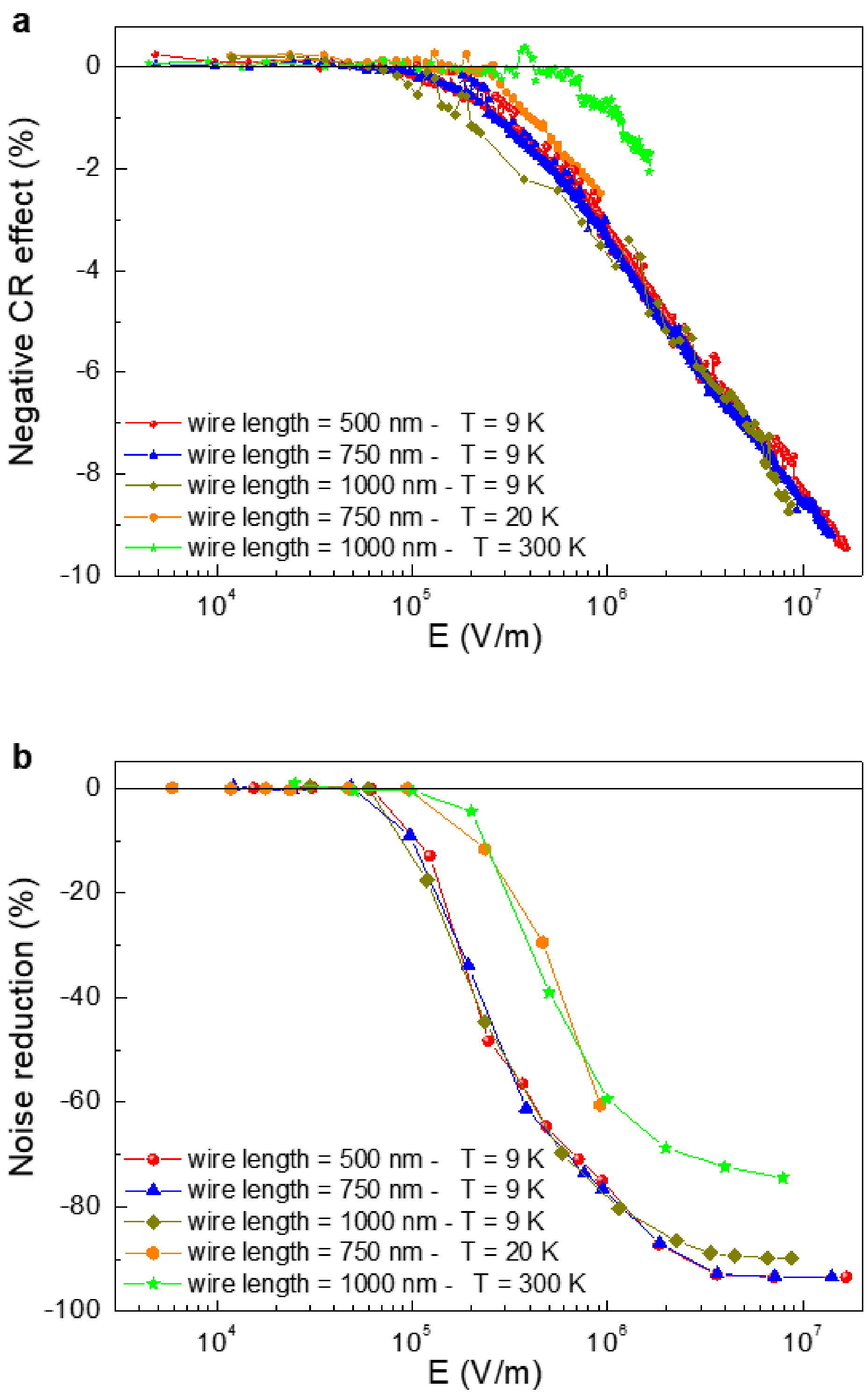

Figure S1. Electric field behavior of resistance and noise data at different temperatures. For the same nanowires as in Figure 8 (main text), it is shown, at different temperatures, the electric field dependence of: (a) the current-resistance effect, evaluated with Equation (2) of the main text, (b) the noise reduction percentage, evaluated with Equation (3) of the main text. The critical electric field E_c seems to be temperature-dependent.
